# Postage on Exchange Medical Journals

**Published:** 1850-03

**Authors:** 


*Postage on Exchange Medical Journals,*—The editor of the Southern Medical and Surgical Journal, in connection with some excellent remarks on the obvious impropriety of subjecting editors of medical periodicals to expense of postage on exchanges, suggests that the editor of every such work should address a memorial to Congress on the subject, and forward it to the Chairman of the Post Office committee. We think the suggestion a good one, and intend to act accordingly. In order that the effort may prove successful, we hope all will unite in so doing.

